# Supplementary material for: A quantitative systems pharmacology model of the pathophysiology and treatment of COVID-19 predicts optimal timing of pharmacological interventions
Source: NPJ Syst Biol Appl. 2023 Apr 14;9:13. doi: 10.1038/s41540-023-00269-6 (PMC10102696; doi:10.1038/s41540-023-00269-6)
Supplement: Supplementary file 1 — Supplementary Materials and Methods [file 41540_2023_269_MOESM1_ESM.pdf]

Supplementary Methods

Overview of model structure

Viral Infection dynamics

We developed a set of ordinary differential equations (ODEs) to describe the dynamics of SARS-CoV-2 viral load, leveraging previously published mathematical models of in-host viral infection dynamics [1-3]. Susceptible alveolar Type II cells (AT2), in our model, are infected by SARS-CoV-2 to form productively infected cells (I), shedding viable virus particles (V). Furthermore, we account for the phenomenological repletion of uninfected alveolar cells due to the activation of wound healing mechanisms [4].

Immune Response Dynamics

Equations from Rogers et al. [5] were adapted to model immune cells activated by free virus, infected cells, and by proinflammatory immune-induced alveolar tissue damage. For simplicity we only consider alveolar and plasma compartments in the model. The key site of infection is represented by the alveolar space, despite the existence of multiple other potentially relevant sites of viral replication and immune activation in COVID-19.

Both free virus and infected cells activate the host immune response in the model [6,7], resulting in the activation or maturation of innate immune cells; alveolar-resident macrophages, neutrophils and dendritic cells (DCs). The dendritic cells are the primary antigen presenting cells (APC) in the model and activate the T-cell populations. The T-cells populations in the model include the CD8+ cytotoxic T-cells (CTL) and the CD4+ Th1, Th17 and Treg cells. While the CD8+ CTLs are involved in the cytotoxic clearance of infected cells, the CD4+ Th1 and Th17 cells support CD8+ T-cell activation through the secretion of proinflammatory cytokines together with DCs, macrophages and neutrophils. The CD4+ Treg cells constitute the primary anti-inflammatory mediators in the model and secrete IL-10 and TGF-β. As an example, Equation 1 describes the activation of CD8+ T-cells as considered in the model.

$$\frac{CTL}{dt} = \alpha_{CTL} DC\left[1 + a_{IFN\beta}\right]\left[1 + a_{IL12}\right]\left[a_{IL10/TGF\beta}\right] - \beta_{CTL} CTL - tr[CTL] \qquad \text{Eq. 1}$$

Where,

$$\text{CTL is the alveolar CD8 + cytotoxic T cells}$$

$$[\text{CTL}](\text{cells}\backslash\mu\text{L}) = \frac{\text{CTL}}{\text{alv\_vol}};$$

$$\text{alv\_vol is the volume of the alveolar compartment}$$

Type I IFN induction of CTL activation

$$a_{IFN\beta} = \frac{k_{MHCI[IFN\beta]} IFN\beta}{km_{MHCI[IFN\beta]} + IFN\beta}$$

IL-12 induction of CTL activation potentiated by IL-2

$$a_{IL12} = \left(\frac{k_{CTL[IL12]} IL12}{km_{CTL[IL12]} + IL12}\right)\left(1 + \frac{k_{CTL[IL2]} IL2}{km_{CTL[IL2]} + IL2}\right)$$

IFN*γ* induction of CTL activation

$$a_{IFN\gamma} = \left(\frac{k_{CTL[IFN\gamma]} IFN\gamma}{km_{CTL[IFN\gamma]} + IFN\gamma}\right)\left(\frac{k_{CTL[IL6]}}{km_{CTL[IL6]} + IL6}\right)$$

IL-10 and TGFβ-mediated inhibition of CTL activation

$$a_{IL10/TGF\beta} = \left(\frac{k_{CTL[IL10]}}{km_{CTL[IL10]} + IL10}\right)\left(\frac{k_{CTL[TGF\beta]}}{km_{CTL[TGF\beta]} + TGF\beta}\right)$$

CD8+ cytotoxic T-cells in the model are activated by viral epitope-responsive DCs. Their activation is further potentiated by the cytokines IL-12, IL-2, interferon (IFN)-$\boldsymbol{\gamma}$, Type I IFN and inhibited by IL-10 and TGF-β. Additionally, the ability of the cytokines IFN-$\boldsymbol{\gamma}$ and IL-6 to negatively regulate each other's activity is also incorporated [8]. The clearance of the CD8+ T-cells is determined by a first-order nonspecific death/deactivation rate (β), and inter-compartmental transport rate ($k_{tr[CTL]}$). Equal immune cell transit rates between alveolar and plasma compartments are assumed for simplicity.

Viral Clearance and Anti-Viral Dynamics

Type I IFN, released by virus-infected cells and mature DCs in the model, inhibits the generation of infected cells by implicitly accounting for effects of IFN-stimulated gene expression products known to confer viral resistance [9]. Additionally, activated CD8+ T-cells contribute to viral clearance through their cytotoxic effects on infected cells, thereby reducing the rate of shedding of infectious virus.

Virus & Immune-induced Damage

To account for the influence of tissue damage underlying the hyperinflammatory pathophysiological outcomes associated with severe COVID-19, we explicitly account for damage and immune-mediated death of alveolar cells that might occur due to both viral infection as well as the effects of proinflammatory cytokines [10,11].

Clinical Biomarkers

The model incorporates three biomarkers commonly monitored in hospitalized cases of COVID-19: C-reactive protein (CRP), which is a general marker of inflammation; and ferritin and surfactant protein D (SP-D), which are leakage products of alveolar cell damage [12,13]. In our model, plasma IL-6 is assumed to induce the production of hepatic CRP, while ferritin and SP-D are released upon the death of damaged alveolar cells. All biomarkers are assumed to be released into systemic circulation from their respective sites of production.

Incorporation of anti-viral and SARS-CoV-2 neutralizing antibody cocktail treatment effects

The anti-viral, molnupiravir is the pro-drug of the pharmacologically active EIDD-1931, a nucleoside analogue which acts by introducing random point mutations throughout the SARS-CoV-2 viral RNA, leading to error catastrophe of viable virus [14]. Informed by this mechanism of action, the pharmacodynamic effects of molnupiravir are modeled as inhibiting the production of viable virus from infected cells. $C_{anti\text{-}viral}$ is the plasma concentration of the anti-viral and IC_{50} is the half-maximal inhibitory concentration of the anti-viral.

$$\frac{dV}{dT} = \alpha_v \cdot I - f_{Vint} k_{int}(AT2) \cdot V \cdot \left(1 - k_{int\_IFN_\beta} \frac{(IFN_\beta)}{km_{int\_IFN_\beta} + (IFN_\beta)}\right) \cdot \left[\frac{Imax.C^p_{anti-viral}}{IC^p_{50} + C^p_{anti-viral}}\right] - \beta_V \cdot V$$

The pharmacodynamic effect of the nAb cocktails are modeled to decrease the rate constant for the production of infected cells due to viable virus. This is informed by their mechanism of action whereby the nAbs selectively bind to the spike protein of SARS-CoV-2, thus neutralizing the virus particles, preventing their entry into susceptible cells, and subsequent replication. The effect of the antibody cocktail in the case of the Blaze-1 trial and REGEN-COV trials is accounted for in an additive manner where the two antibodies we assume at high combination doses each nAb contributes equally to efficacy. This additive incorporation is in agreement with the pharmacodynamic modeling performed by the investigators of the Blaze-1 trial in [15,16]. $CONC_1$ and $CONC_2$ are the plasma concentrations and $IC_{1,50}$ and $IC_{2,50}$ are the half-maximal inhibitory concentration of each of the antibodies comprising the neutralizing antibody cocktail.

$$\frac{dI}{dT} = k_{int}(AT2) \cdot V \left(1 - k_{int(IFN_\beta)} \frac{(IFN_\beta)}{km_{int\_IFN_\beta} + (IFN_\beta)}\right) \cdot \left[1 - Imax_{nab}\left[\frac{CONC_1^p}{IC_{1,50}^p + CONC_1^p} + \frac{CONC_2^p}{IC_{2,50}^p + CONC_2^p}\right]\right] - \beta_I \cdot I - k_{kill}\left(1 + \frac{(IFN_\beta)}{km_{kill} + (IFN_\beta)}\right) \cdot I \cdot (CTL) - k_{ROS\_damage} \cdot I$$

We note that the IC_{50} of the antibodies are specific to the wild-type variant of SARS-CoV-2, informed by the lineage of SARS-CoV-2 prevalent when these trials were performed. However, the IC_{50} can be appropriately updated for specific variants of concern (VOC) if needed to simulate the impact of VOC on the clinical efficacy of these therapeutics. The IC_{50} of the anti-viral molnupiravir was found to not be attenuated for the VOC, such as the Omicron variant [17].

Supplementary Figures

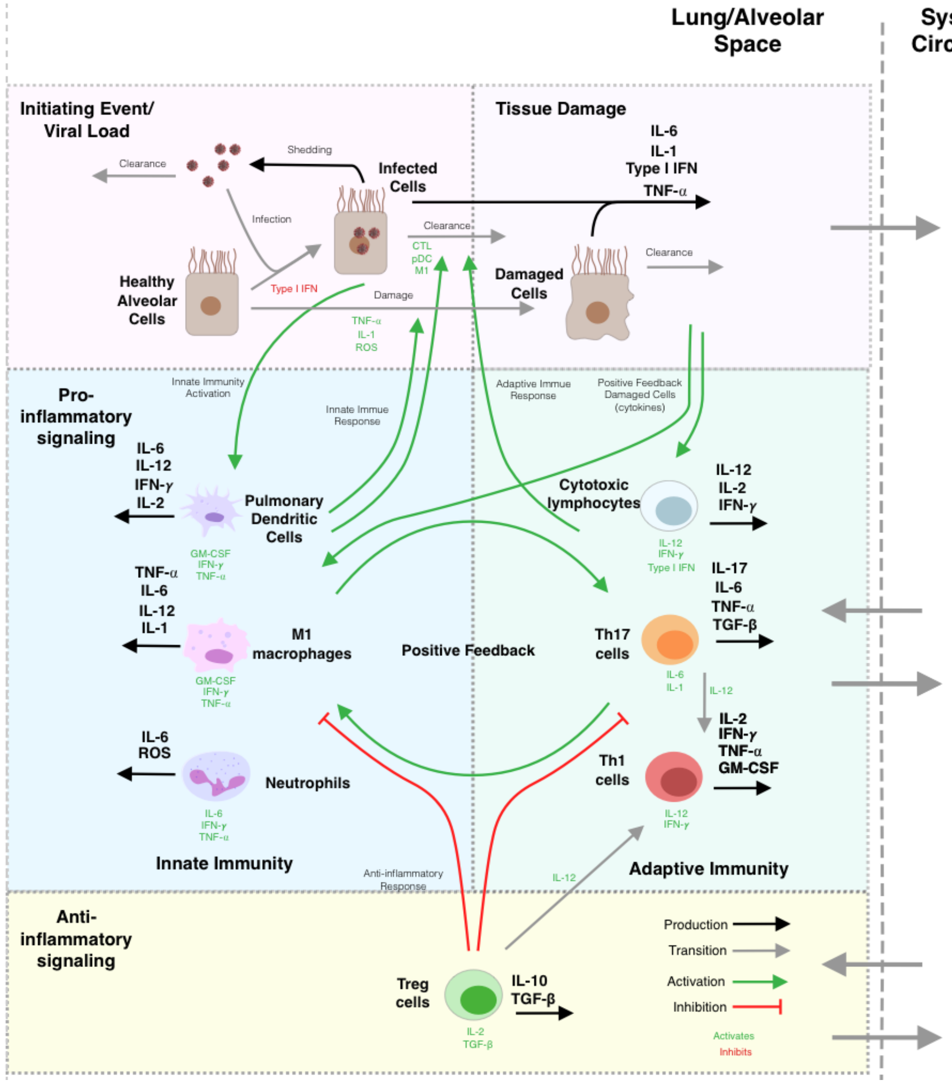

Supplementary Figure 1: Detailed schematic of mechanistic interactions accounted for in the COVID-19 QSP model. This schematic is reproduced from Dai et al. [18]

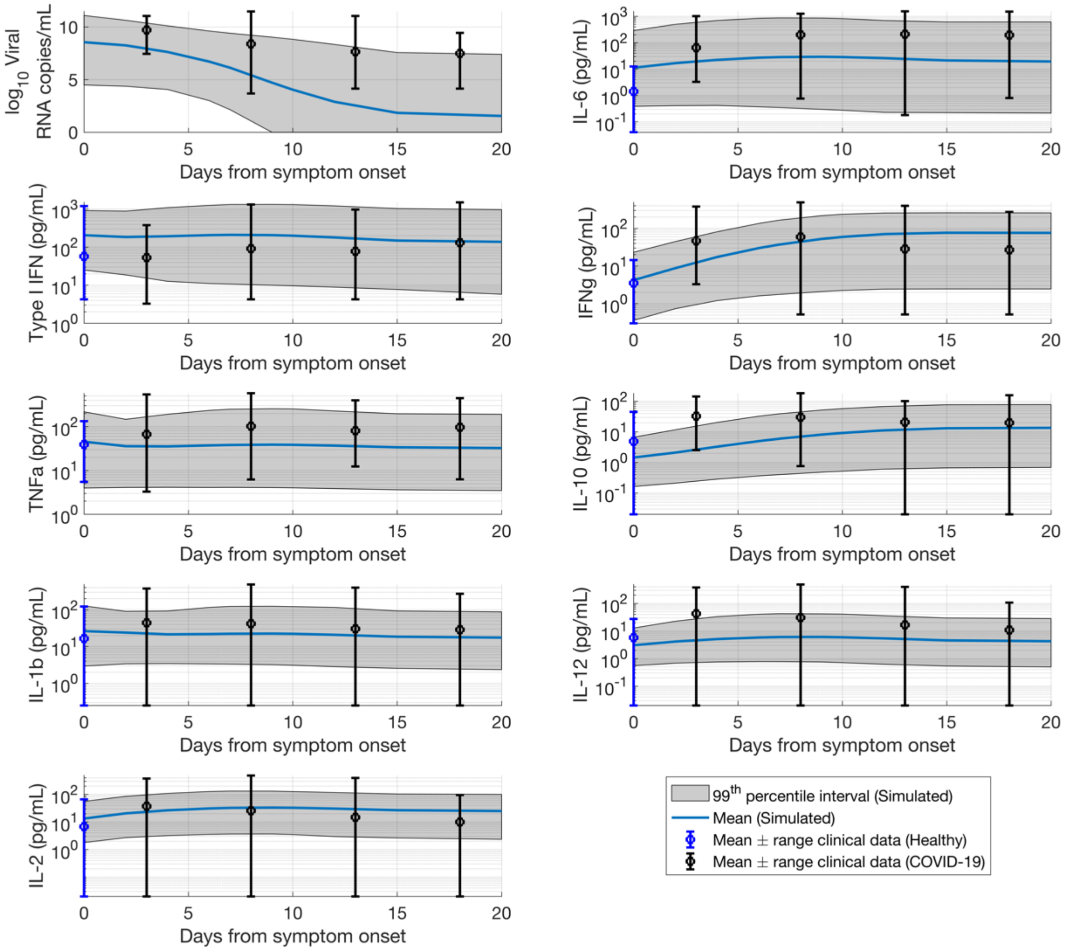

Supplementary Figure 2: Plausible population (N = 14545) overlaid against observational COVID-19 clinical data for the viral load time course and different representative cytokines. The time course is presented relative to days from symptom onset given the assumption the symptom onset coincides with viral load peak for each virtual subject. Data extracted from studies listed in Supplementary Table S1.

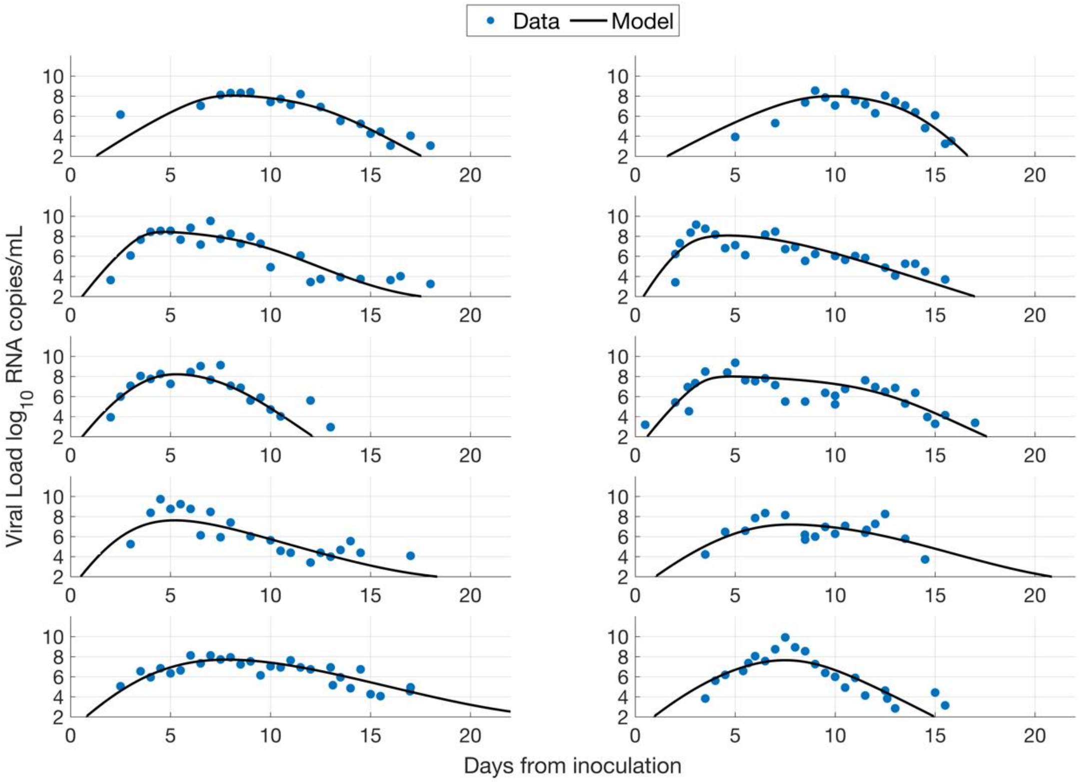

Supplementary Figure 3: Virtual subjects selected from the plausible population to match the individual viral load time courses from inoculation to viral clearance from participants in a SARS-CoV-2 human challenge study detailed in Killingley et al [19], thus showing that the plausible population is composed of subjects that exhibit viral dynamics that are physiologically realistic. The threshold for inactive virus is 10^2 RNA copies/mL comparable to the LLOQ [58 RNA copies/mL] for the PCR assay used in [19]. Data are extracted for subjects with confirmed symptomatic SARS-CoV-2 infection with PCR assay measurements above the limit of quantification upon viral inoculation from [19].

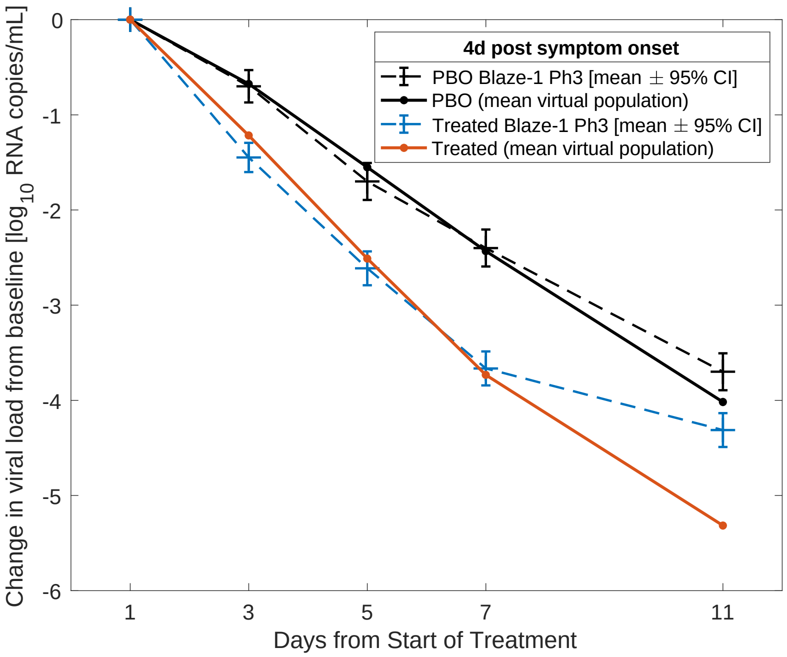

Supplementary Figure 4: Mean change in viral load from baseline for the Blaze-1 virtual population under placebo (black solid line) and treated (orange solid line) conditions compared against the reported clinical observations (mean±95% CI) in the Blaze-1 Ph3 trial for placebo conditions (black dashed) and upon treatment with 2800mg bamlanivimab and 2800mg etesevimab (blue dashed). Data extracted from Dougan et al. [20].

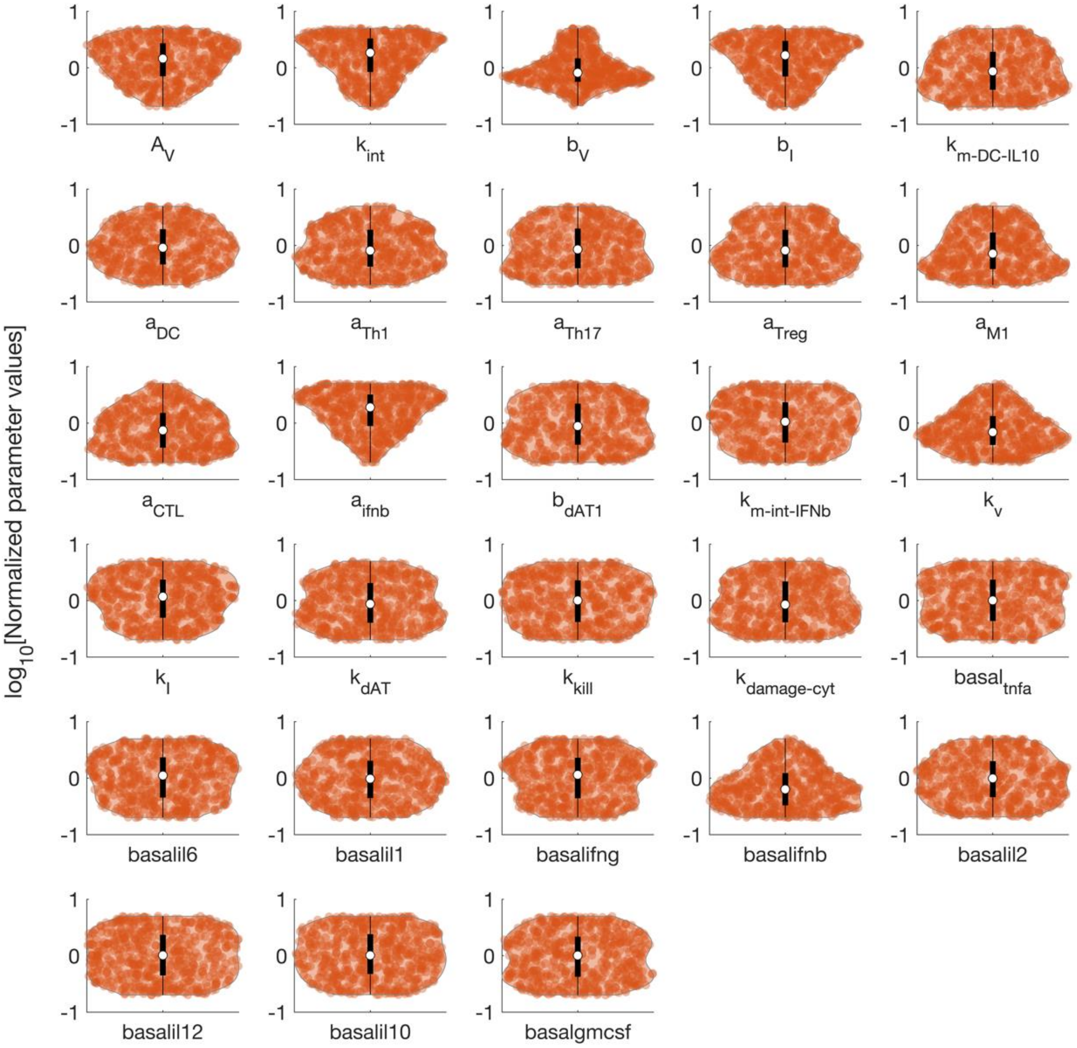

Supplementary Figure 5: Distributions of Blaze-1 virtual population parameters normalized by the nominal value of each parameter

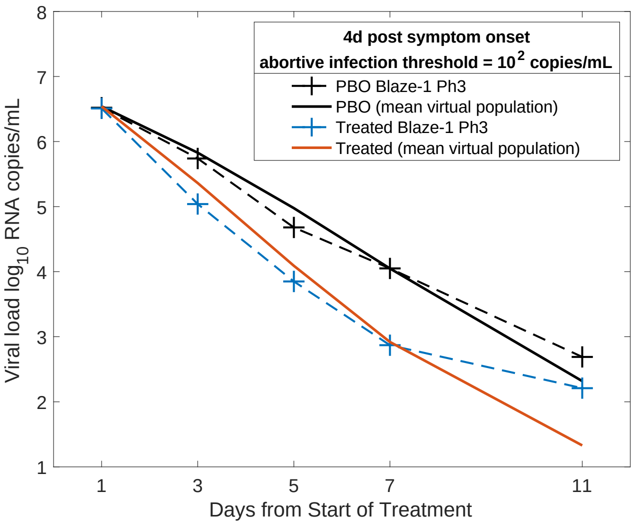

Supplementary Figure 6: Virtual population matching the observations from the Blaze-1 Ph3 trial where the threshold for inactive virus is 10^2 RNA copies/mL. Mean of the virtual population (N=502) for the simulated placebo (PBO) group and the 2800mg bamlanivimab + 2800mg etesevimab simulated treated group matching the mean trial data from the observed Blaze-1 Ph3 placebo group and the 2800mg bamlanivimab + 2800mg etesevimab treated group. Data extracted from [21].

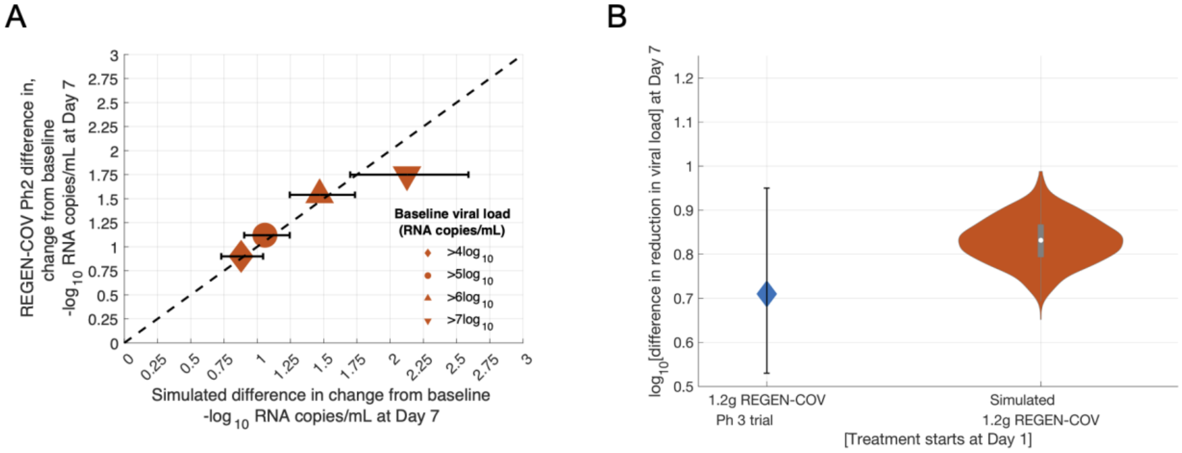

Supplementary Figure 7: A) $\log_{10}$ reduction in viral load from baseline at Day 7 for the overall virtual population and each of the subgroups compared against observations from the REGEN-COV Ph2 clinical trial for the placebo group and the 8g REGEN COV treatment group. Error bars are representative of the 99% prediction interval of the mean for the virtual population B) $\log_{10}$ reduction in viral load from baseline at Day 7 for the 1.2g REGEN-COV treatment group. Data extracted from [22].

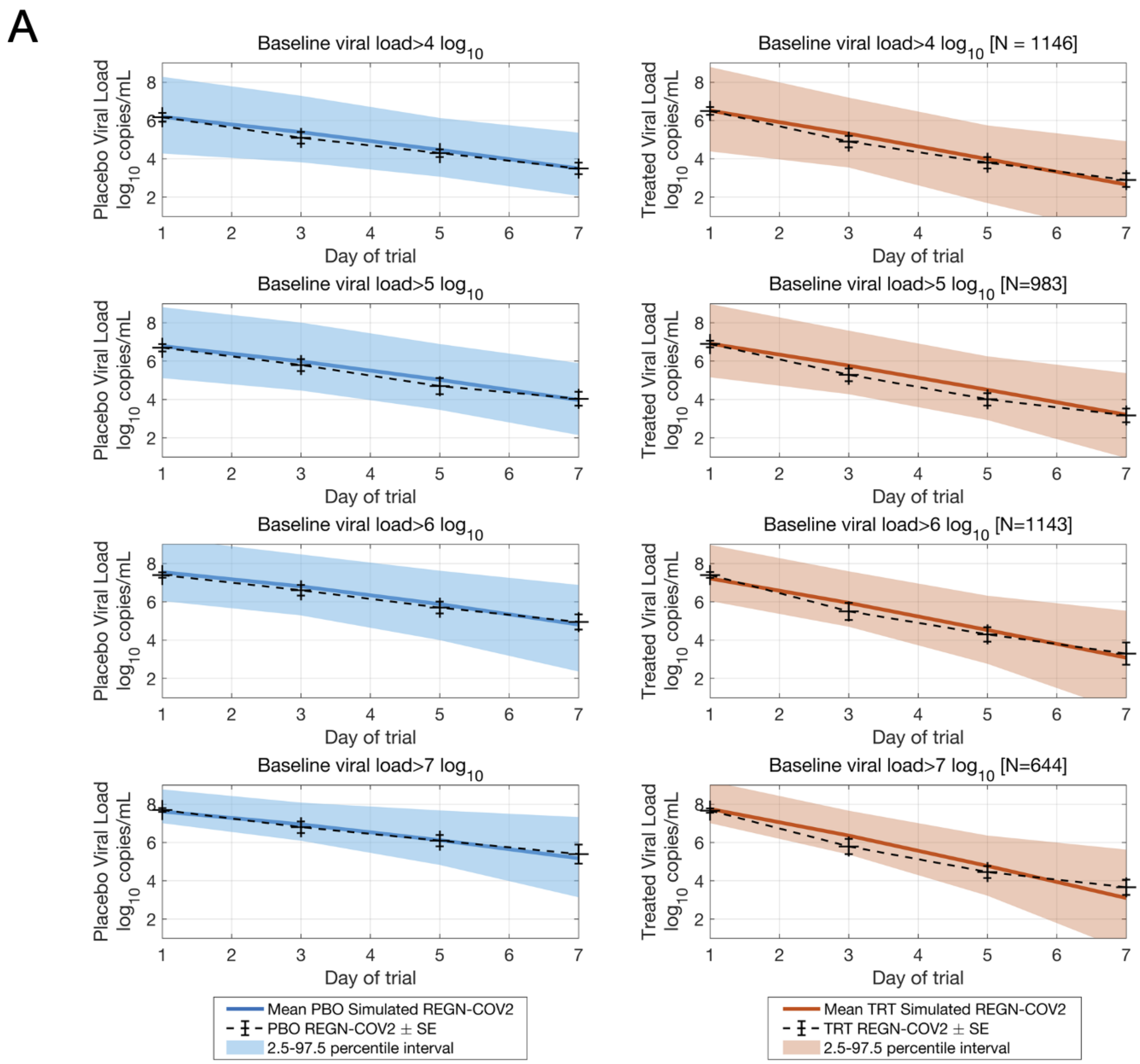

A
Baseline viral load>4 log10
Baseline viral load>4 log10 [N = 1146]
Baseline viral load>5 log10
Baseline viral load>5 log10 [N=983]
Baseline viral load>6 log10
Baseline viral load>6 log10 [N=1143]
Baseline viral load>7 log10
Baseline viral load>7 log10 [N=644]
Placebo Viral Load log10 copies/mL
Treated Viral Load log10 copies/mL
Day of trial
Mean PBO Simulated REGN-COV2
PBO REGN-COV2 ± SE
2.5-97.5 percentile interval
Mean TRT Simulated REGN-COV2
TRT REGN-COV2 ± SE
2.5-97.5 percentile interval

114

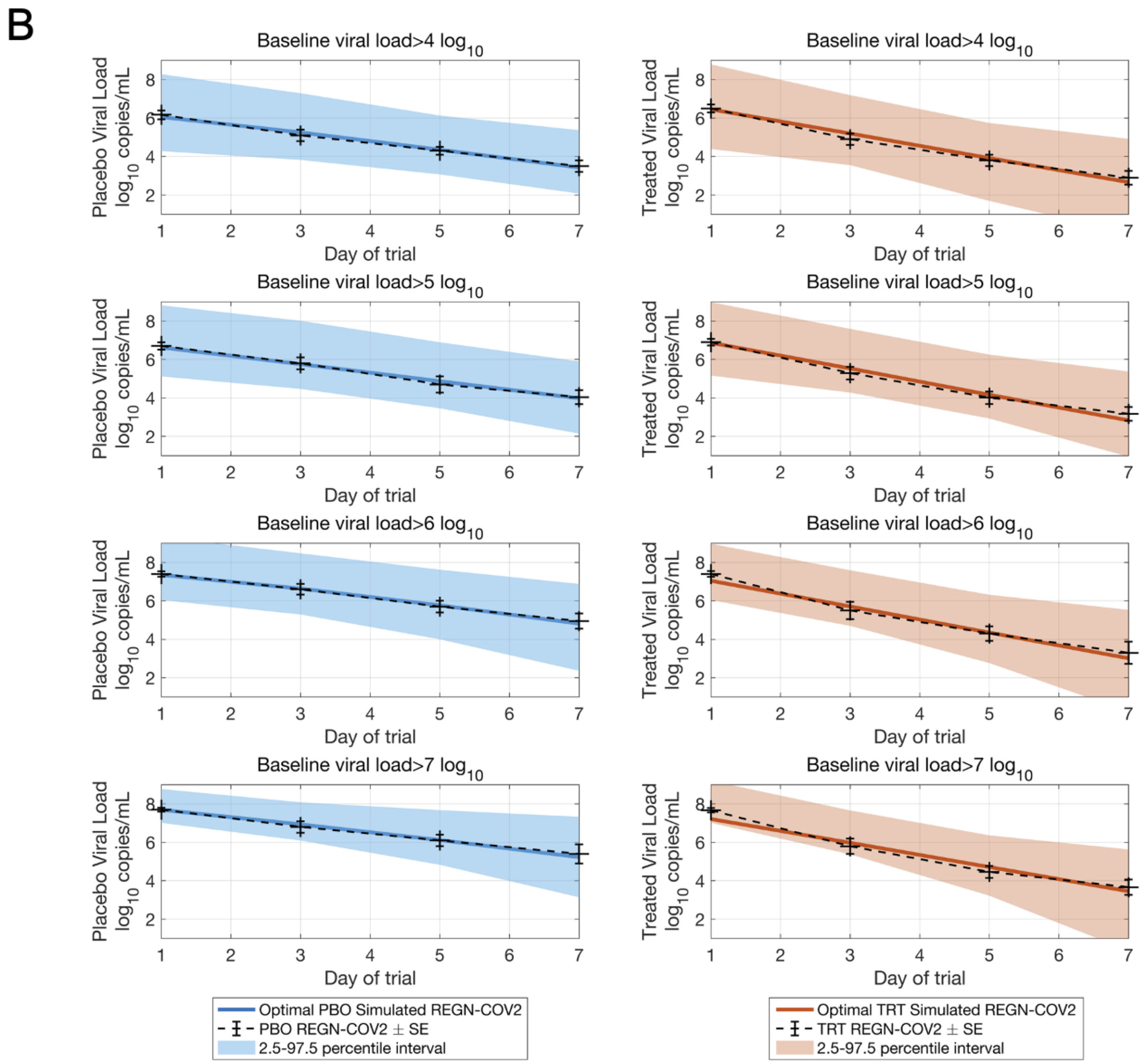

Supplementary Figure 8: Virtual populations selected from the plausible population to match the subgroups by baseline viral load presented in the REGEN-COV Ph2 study. Mean viral load trajectories for each subgroup are shown in dashed black lines with as extracted from [22], the shaded regions represent the 95% intervals for the simulated placebo (blue) and treated group (orange) respectively, the solid lines represent A) the mean of the virtual populations and B) the virtual subject from the plausible population that most closely matches the mean viral load dynamics for each subgroup.

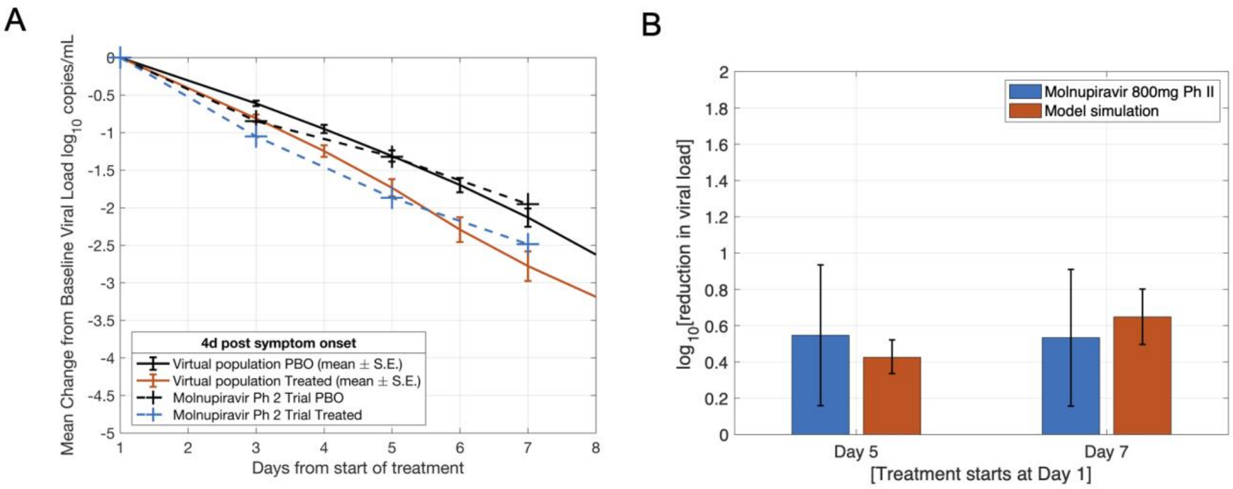

Supplementary Figure 9: Virtual population matched to recapitulate A) the observed time course of the mean change in viral load from baseline for the placebo and treated groups upon administration of 800mg Molnupiravir BID, B) the reduction in viral load from baseline at Day 5 and Day 7 from treatment. Data extracted from [23].

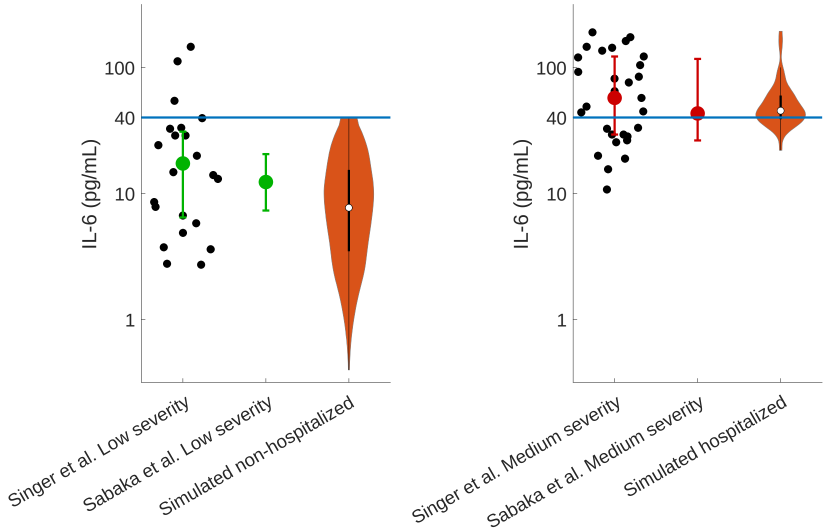

Supplementary Figure 10: The IL-6 threshold of 40pg/ml is in qualitative agreement with observations of plasma IL-6 levels in COVID-19 patients with mild disease severity (outpatient COVID-19 subjects or hospitalized patients but without the hypoxemia/need for oxygen therapy) and COVID-19 patients with moderate to severe disease severity (hospitalized patients in need of oxygen support) as reported in [24,25], respectively. Simulated IL-6 levels are pooled and plotted at Days 5-11 post symptom onset in simulated COVID-19 infection, so as to be comparable to the baseline demographics of the patient populations in these studies.

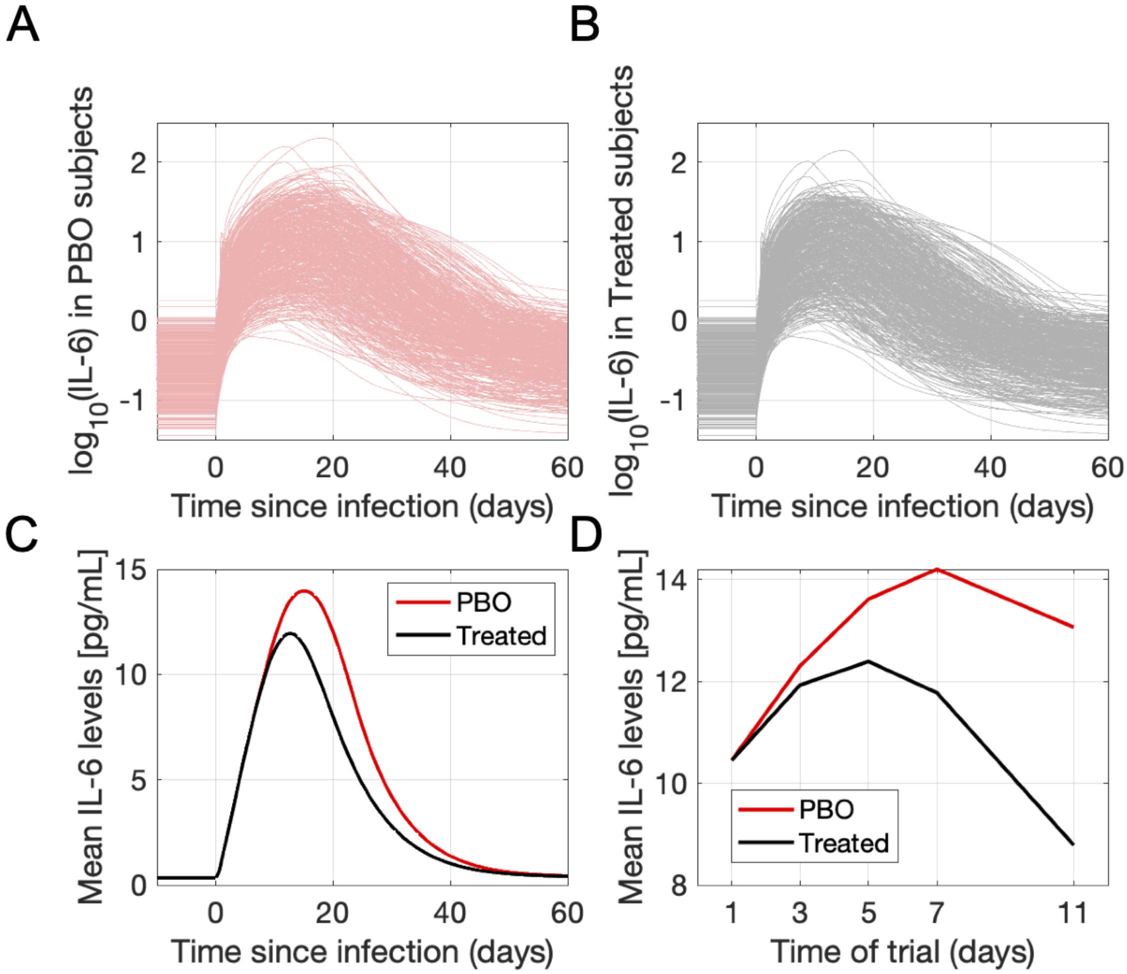

Supplementary Figure 11: A & B) Placebo and treated IL-6 time courses for the individual virtual subjects making up the Blaze-1 virtual population, respectively. c) the mean IL-6 time course from time since infection for the Blaze-1 virtual population in placebo and treated conditions d) The mean IL-6 time course from time since start of the trial for the Blaze-1 virtual population in placebo and treated conditions.

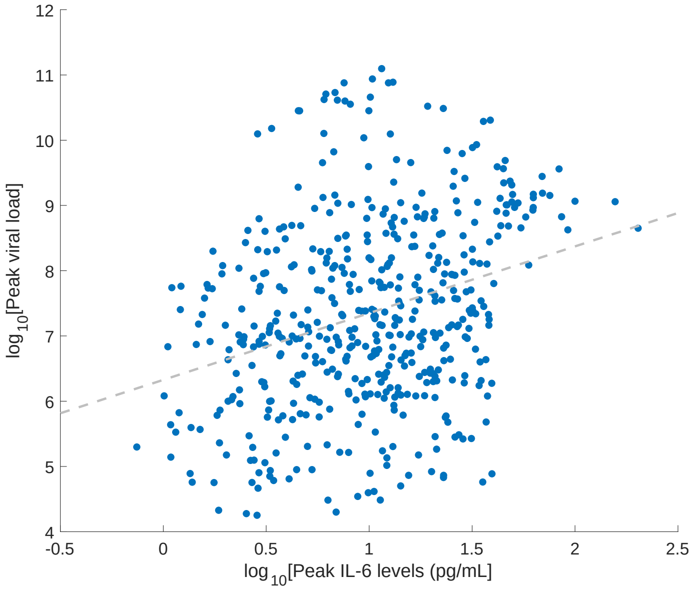

Supplementary Figure 12: Correlation between peak viral load and peak IL-6 levels for Blaze-1 virtual population (Correlation coefficient = 0.31).

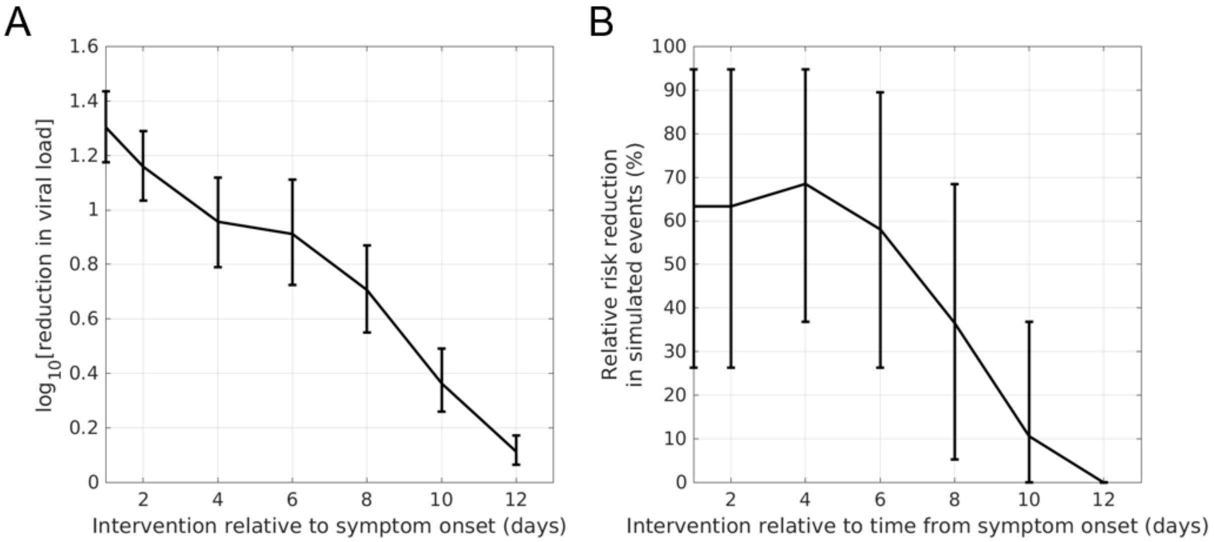

Supplementary Figure 13: A) Virological and B) clinical efficacy exhibiting similar sensitivity to the time of intervention after treatment with REGEN-COV nAb cocktail.

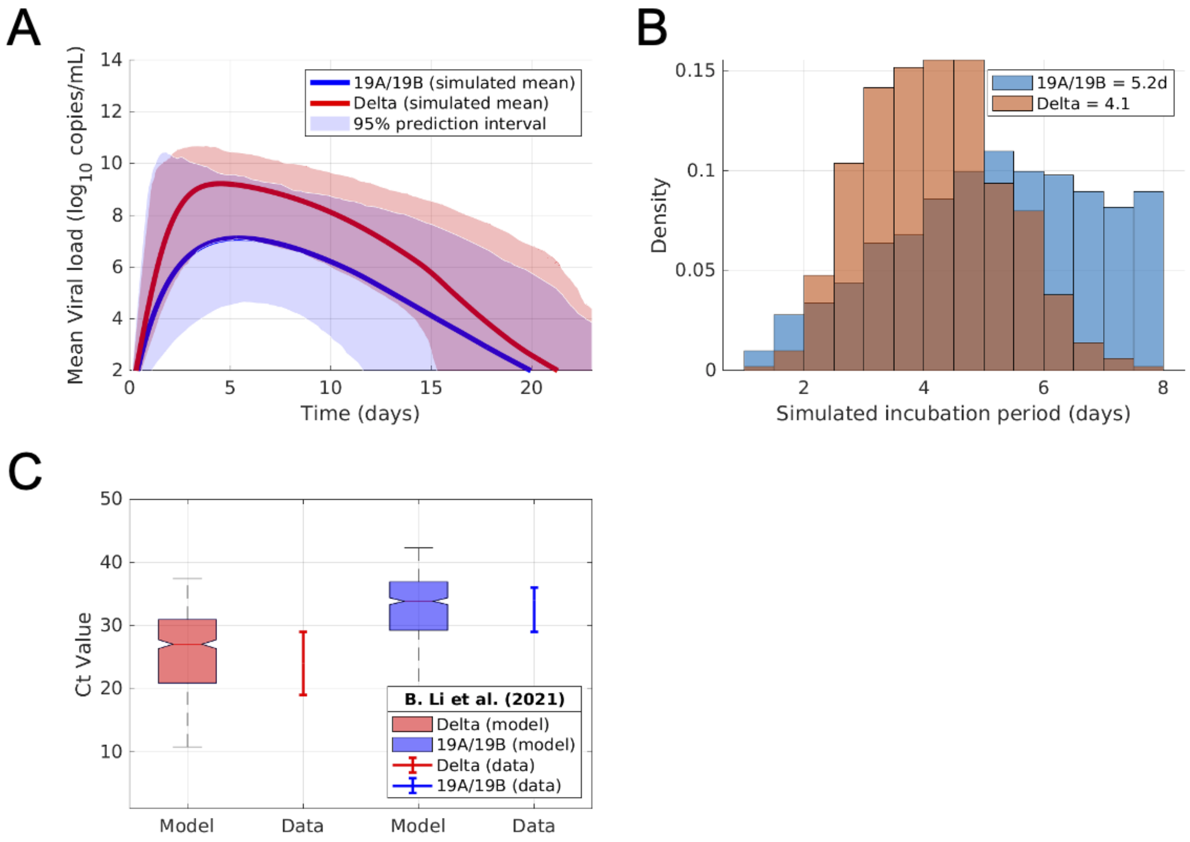

Supplementary Figure 14: Virtual population with delta variant viral dynamics. A) Li et al. provided viral load measurements enabling an approximate conversion between PCR assay Ct values and RNA copies/mL. Virtual subjects were selected from the previously developed plausible population to match the reported viral load measurements in Li et al, assuming the viral dynamics from the RCT-matched virtual population were equivalent to the viral dynamics of the reported 19A/19B clade and assuming the PCRs assays were performed ~2d post infection for both the 19A/19B clade and Delta variant. B) The simulated incubation period for the Delta variant and 19A/19B clade virtual populations C) The simulated viral load time course of the Delta variant virtual population (red) compared to the non-delta SARS-CoV-2 clades prevalent in 2019-2020. Data extracted from [26].

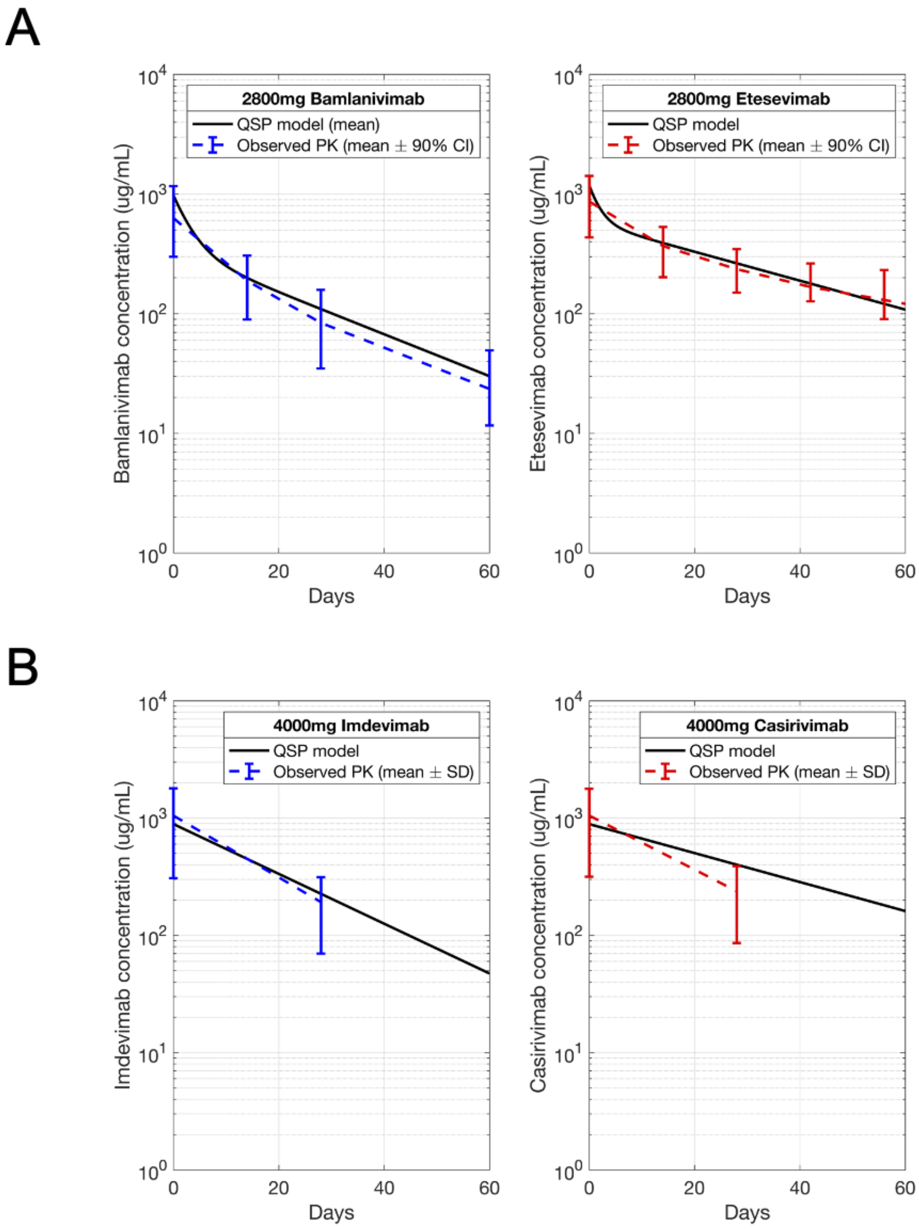

Supplementary Figure 15: Observed and simulated plasma concentration profiles for A) 2800mg bamlanivimab and 2800mg etesevimab from the Blaze-1 Ph3 trial B) 4000mg imdevimab and 4000mg casirivimab from the REGEN-COV Ph2 trial. Data extracted from [15] and [22].

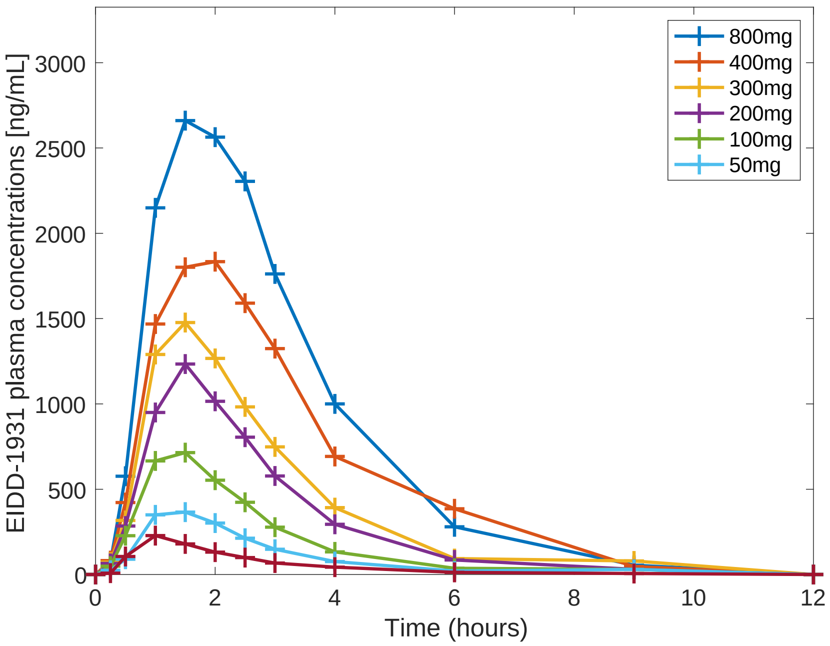

Supplementary Figure 16: Plasma concentration profile of EIDD-1931 (active metabolite of molnupiravir) extracted from Painter et al. [1]. The digitized points are indicated with + symbols.

Supplementary Table 1: Datasets used to construct plausible population

	References	State	Population
1	Lucas, C.; Wong, P.; Klein, J.; Castro, T.B.R.; Silva, J.; Sundaram, M.; Ellingson, M.K.; Mao, T.; Oh, J.E.; Israelow, B., et al. Longitudinal analyses reveal immunological misfiring in severe COVID-19. *Nature* **2020**, *584*, 463-469, doi:10.1038/s41586-020-2588-y.	Tumor necrosis factor-α, Interleukin-6, Interleukin-1β, Type I Interferon [Interferon-β], Interferon-***γ***, Interleukin-12, Interleukin-2, Interleukin-10	Hospitalized COVID-19 patients
2	Mudd, P.A.; Crawford, J.C.; Turner, J.S.; Souquette, A.; Reynolds, D.; Bender, D.; Bosanquet, J.P.; Anand, N.J.; Striker, D.A.; Martin, R.S., et al. Distinct inflammatory profiles distinguish COVID-19 from influenza with limited contributions from cytokine storm. *Sci Adv* **2020**, *6*, doi:10.1126/sciadv.abe3024.	Granulocyte-maturation colony stimulating factor, dendritic cells, CD8+ T-cells, CD4+ T-cells in hospitalized patients	Hospitalized COVID-19 patients
3	Mann, E.R.; Menon, M.; Knight, S.B.; Konkel, J.E.; Jagger, C.; Shaw, T.N.; Krishnan, S.; Rattray, M.; Ustianowski, A.; Bakerly, N.D., et al. Longitudinal immune profiling reveals key myeloid signatures associated with COVID-19. *Sci Immunol* **2020**, *5*, doi:10.1126/sciimmunol.abd6197.	Interleukin-6, Interleukin-10 in hospitalized patients	Hospitalized COVID-19 patients
4	Gastine, S. et al. Systematic Review and Patient-Level Meta-Analysis of SARS-CoV-2 Viral Dynamics to Model Response to Antiviral Therapies. Clin Pharmacol Ther 110, 321-333, doi:10.1002/cpt.2223 (2021).	Viral load	COVID-19 outpatients and hospitalized COVID-19 patients
5	Van Singer, M., Brahier, T., Ngai, M., Wright, J., Weckman, A.M., Erice, C., Meuwly, J.Y., Hugli, O., Kain, K.C. and Boillat-Blanco, N.,. COVID-19 risk stratification algorithms based on sTREM-1 and IL-6 in emergency department. *Journal of Allergy and Clinical Immunology, 147(1), pp.99-106.* **2021**	IL-6	COVID-19 outpatients and hospitalized COVID-19 patients
6	Sabaka, P., Koščálová, A., Straka, I., Hodosy, J., Lipták, R., Kmotorková, B., Kachlíková, M. and Kušnírová, A.,. Role of interleukin 6 as a predictive factor for a severe course of Covid-19: retrospective data analysis of patients from a long-term care facility during Covid-19 outbreak. *BMC infectious diseases, 21(1), pp.1-8.* **2021**	IL-6	COVID-19 outpatients and hospitalized COVID-19 patients

Supplementary Table 2: Parameters varied to generate plausible population

Parameter	Description
A_V	viral shedding by infected cells
b_V	endogenous viral clearance
b_I	death rate for infected cells
a_DC	rate constant for production of mature dendritic cells
km_DC_IL10	IC50 for inhibition of DC activation by IL-10
a_Th1	rate constant for activation of Th1 cells
a_Th17	rate constant for activation of Th17 cells
a_Treg	rate constant for Treg activation
a_M1	rate constant for activation of macrophages
a_CTL	rate constant for CTL activation
a_ifnb	basal induction of Type I IFN
b_dAT1	death rate for damaged AT1 cells
km_int_IFNb	IC50 for anti-viral effects of Type I IFN
k_v	rate constant for viral activation of innate immune cells
k_I	rate constant for innate immune activation by infected cells
k_dAT	rate constant for innate immune activation by damaged cells
k_kill	rate constant for infected cell clearance by CD8+ cell clearance
k_damage_cyt	rate constant overall cytokine damage
k_int	viral endocytosis by AT2
basal_tnfa	basal production rate of TNF
basalil6	basal production rate of IL-6
basalil1	basal production rate of IL-1
basalifng	basal production rate of IFNg
basalifnb	basal production rate of Type I IFN
basalil2	basal production rate of IL-2
basalil12	basal production of IL-12
basalgmcsf	basal production of GM-CSF
basalil10	basal production rate of IL-10

Supplementary References

1. Painter, W.P.; Holman, W.; Bush, J.A.; Almazedi, F.; Malik, H.; Eraut, N.; Morin, M.J.; Szewczyk, L.J.; Painter, G.R. Human Safety, Tolerability, and Pharmacokinetics of Molnupiravir, a Novel Broad-Spectrum Oral Antiviral Agent with Activity Against SARS-CoV-2. *Antimicrob Agents Chemother* **2021**, 10.1128/AAC.02428-20, doi:10.1128/AAC.02428-20.

1. Baccam, P.; Beauchemin, C.; Macken, C.A.; Hayden, F.G.; Perelson, A.S. Kinetics of influenza A virus infection in humans. *J Virol* **2006**, *80*, 7590-7599, doi:10.1128/JVI.01623-05.
2. Lee, H.Y.; Topham, D.J.; Park, S.Y.; Hollenbaugh, J.; Treanor, J.; Mosmann, T.R.; Jin, X.; Ward, B.M.; Miao, H.; Holden-Wiltse, J., et al. Simulation and prediction of the adaptive immune response to influenza A virus infection. *J Virol* **2009**, *83*, 7151-7165, doi:10.1128/JVI.00098-09.

3. Pawelek, K.A.; Huynh, G.T.; Quinlivan, M.; Cullinane, A.; Rong, L.; Perelson, A.S. Modeling within-host dynamics of influenza virus infection including immune responses. *PLoS Comput Biol* **2012**, *8*, e1002588, doi:10.1371/journal.pcbi.1002588.
4. Herold, S.; Becker, C.; Ridge, K.M.; Budinger, G.R. Influenza virus-induced lung injury: pathogenesis and implications for treatment. *Eur Respir J* **2015**, *45*, 1463-1478, doi:10.1183/09031936.00186214.
5. Palsson, S.; Hickling, T.P.; Bradshaw-Pierce, E.L.; Zager, M.; Jooss, K.; O'Brien, P.J.; Spilker, M.E.; Palsson, B.O.; Vicini, P. The development of a fully-integrated immune response model (FIRM) simulator of the immune response through integration of multiple subset models. *BMC Syst Biol* **2013**, *7*, 95, doi:10.1186/1752-0509-7-95.
6. Iwasaki, A.; Medzhitov, R. Control of adaptive immunity by the innate immune system. *Nat Immunol* **2015**, *16*, 343-353, doi:10.1038/ni.3123.
7. Iwasaki, A.; Pillai, P.S. Innate immunity to influenza virus infection. *Nat Rev Immunol* **2014**, *14*, 315-328, doi:10.1038/nri3665.
8. Wormald, S.; Zhang, J.G.; Krebs, D.L.; Mielke, L.A.; Silver, J.; Alexander, W.S.; Speed, T.P.; Nicola, N.A.; Hilton, D.J. The comparative roles of suppressor of cytokine signaling-1 and -3 in the inhibition and desensitization of cytokine signaling. *J Biol Chem* **2006**, *281*, 11135-11143, doi:10.1074/jbc.M509595200.
9. Perry, A.K.; Chen, G.; Zheng, D.; Tang, H.; Cheng, G. The host type I interferon response to viral and bacterial infections. *Cell Res* **2005**, *15*, 407-422, doi:10.1038/sj.cr.7290309.
10. Tisoncik, J.R.; Korth, M.J.; Simmons, C.P.; Farrar, J.; Martin, T.R.; Katze, M.G. Into the eye of the cytokine storm. *Microbiol Mol Biol Rev* **2012**, *76*, 16-32, doi:10.1128/MMBR.05015-11.
11. Moore, J.B.; June, C.H. Cytokine release syndrome in severe COVID-19. *Science* **2020**, *368*, 473-474, doi:10.1126/science.abb8925.
12. Garcia-Laorden, M.I.; Lorente, J.A.; Flores, C.; Slutsky, A.S.; Villar, J. Biomarkers for the acute respiratory distress syndrome: how to make the diagnosis more precise. *Ann Transl Med* **2017**, *5*, 283, doi:10.21037/atm.2017.06.49.
13. Capelozzi, V.L.; Allen, T.C.; Beasley, M.B.; Cagle, P.T.; Guinee, D.; Hariri, L.P.; Husain, A.N.; Jain, D.; Lantuejoul, S.; Larsen, B.T., et al. Molecular and Immune Biomarkers in Acute Respiratory Distress Syndrome: A Perspective From Members of the Pulmonary Pathology Society. *Arch Pathol Lab Med* **2017**, *141*, 1719-1727, doi:10.5858/arpa.2017-0115-SA.
14. Sheahan, T.P.; Sims, A.C.; Zhou, S.; Graham, R.L.; Pruijssers, A.J.; Agostini, M.L.; Leist, S.R.; Schafer, A.; Dinnon, K.H., 3rd; Stevens, L.J., et al. An orally bioavailable broad-spectrum antiviral inhibits SARS-CoV-2 in human airway epithelial cell cultures and multiple coronaviruses in mice. *Sci Transl Med* **2020**, *12*, doi:10.1126/scitranslmed.abb5883.
15. Emergency Use Authorization (EUA) for Bamlanivimab 700 mg and Etesevimab 1400 mg IV Administered Together Center for Drug Evaluation and Research (CDER) Review. 131.
16. Chigutsa, E.; O'Brien, L.; Ferguson-Sells, L.; Long, A.; Chien, J. Population Pharmacokinetics and Pharmacodynamics of the Neutralizing Antibodies Bamlanivimab and Etesevimab in Patients With Mild to Moderate COVID-19 Infection. *Clin Pharmacol Ther* **2021**, *110*, 1302-1310, doi:10.1002/cpt.2420.
17. Li, P.; Wang, Y.; Lavrijsen, M.; Lamers, M.M.; de Vries, A.C.; Rottier, R.J.; Bruno, M.J.; Peppelenbosch, M.P.; Haagmans, B.L.; Pan, Q. SARS-CoV-2 Omicron variant is highly sensitive to molnupiravir, nirmatrelvir, and the combination. *Cell Res* **2022**, *32*, 322-324, doi:10.1038/s41422-022-00618-w.
18. Dai, W.; Rao, R.; Sher, A.; Tania, N.; Musante, C.J.; Allen, R. A Prototype QSP Model of the Immune Response to SARS-CoV-2 for Community Development. *CPT Pharmacometrics Syst Pharmacol* **2020**, 10.1002/psp4.12574, doi:10.1002/psp4.12574.

19. Killingley, B.; Mann, A.J.; Kalinova, M.; Boyers, A.; Goonawardane, N.; Zhou, J.; Lindsell, K.; Hare, S.S.; Brown, J.; Frise, R., et al. Safety, tolerability and viral kinetics during SARS-CoV-2 human challenge in young adults. *Nat Med* **2022**, *28*, 1031-1041, doi:10.1038/s41591-022-01780-9.
20. Dougan, M.; Nirula, A.; Azizad, M.; Mocherla, B.; Gottlieb, R.L.; Chen, P.; Hebert, C.; Perry, R.; Boscia, J.; Heller, B., et al. Bamlanivimab plus Etesevimab in Mild or Moderate Covid-19. *N Engl J Med* **2021**, *385*, 1382-1392, doi:10.1056/NEJMoa2102685.
21. Eli Lilly. SARS-CoV-2 Neutralizing Antibody Program Update https://investor.lilly.com/static-files/081a5ef7-f5d6-4acc-b0d2-7ae4daf9e953. 26 January 2021.
22. Weinreich, D.M.; Sivapalasingam, S.; Norton, T.; Ali, S.; Gao, H.; Bhore, R.; Musser, B.J.; Soo, Y.; Rofail, D.; Im, J., et al. REGN-COV2, a Neutralizing Antibody Cocktail, in Outpatients with Covid-19. *N Engl J Med* **2021**, *384*, 238-251, doi:10.1056/NEJMoa2035002.
23. Painter, W.P.; Holman, W.; Bush, J.A.; Almazedi, F.; Malik, H.; Eraut, N.; Morin, M.J.; Szewczyk, L.J.; Painter, G.R. Human Safety, Tolerability, and Pharmacokinetics of Molnupiravir, a Novel Broad-Spectrum Oral Antiviral Agent with Activity Against SARS-CoV-2. *Antimicrob Agents Chemother* **2021**, 10.1128/AAC.02428-20, doi:10.1128/AAC.02428-20.
24. Van Singer, M.; Brahier, T.; Ngai, M.; Wright, J.; Weckman, A.M.; Erice, C.; Meuwly, J.Y.; Hugli, O.; Kain, K.C.; Boillat-Blanco, N. COVID-19 risk stratification algorithms based on sTREM-1 and IL-6 in emergency department. *J Allergy Clin Immunol* **2021**, *147*, 99-106 e104, doi:10.1016/j.jaci.2020.10.001.
25. Sabaka, P.; Koscalova, A.; Straka, I.; Hodosy, J.; Liptak, R.; Kmotorkova, B.; Kachlikova, M.; Kusnirova, A. Role of interleukin 6 as a predictive factor for a severe course of Covid-19: retrospective data analysis of patients from a long-term care facility during Covid-19 outbreak. *BMC Infect Dis* **2021**, *21*, 308, doi:10.1186/s12879-021-05945-8.
26. Li, B.; Deng, A.; Li, K.; Hu, Y.; Li, Z.; Xiong, Q.; Liu, Z.; Guo, Q.; Zou, L.; Zhang, H., et al. *Viral infection and transmission in a large, well-traced outbreak caused by the SARS-CoV-2 Delta variant*; 2021/07/23/, 2021; p 2021.2007.2007.21260122.

Model Equations

1 Virus (V)

$$\frac{dV}{dT} = \alpha_v \cdot I - f_{Vint} k_{int}(AT2) \cdot V \cdot \left(1 - k_{int\_IFN_\beta} \frac{(IFN_\beta)}{km_{int\_IFN_\beta} + (IFN_\beta)}\right) - \beta_V \cdot V$$

The virus is infects healthy type II alveolar cells $(AT2)$ and is productively shed by these infected cells I. Type I Interferon $(IFN\beta)$ inihibits the formation of infected cells through an indirect response model. The virus is can also be phagocytosed by dendritic cells (DC) and macrophages $(M1)$ and is undergoes non-specific clearance at a rate β_V.

2 Healthy Alveolar Type 2 Cell $(AT2)$

$$\frac{dAT2}{dT} = \mu_{AT2}(AT2) - k_{int}(AT2) \cdot V \cdot \left(1 - k_{int[IFN_\beta)} \frac{(IFN_\beta)}{km_{int\_IFN_\beta} + (IFN_\beta)}\right) - \beta_{AT2}(AT2) - k_{ROS\_damage}(AT2) - k_{cyt\_damage}(AT$$

$$\mu_{AT2} = \mu_{basal_{[AT2)}} \left(1 + k_\mu \frac{\delta AT}{km_\mu + \delta AT}\right)$$

$$\delta AT2 = AT1_{basal} + AT2_{basal} - (AT1 + AT2)$$

$$k_{cyt\_damage} = k_{damage}\left[\left(\frac{k_{damage\_TNF}(TNF)}{km_{damage\_TNF} + (TNF)}\right) + \frac{k_{damage\_IL6}(IL6)}{km_{damage\_IL6} + (IL6)} + \frac{k_{damage\_IL1\beta}(IL1\beta)}{km_{damage\_IL1\beta} + (IL1\beta)} + \frac{k_{damage\_IFN\gamma}(IFN\gamma)}{km_{damage\_IFN\gamma} + (IFN\gamma)}\right]$$

$$k_{ROS\_damage} = k_{ROS\_N}\left(\frac{N}{km_{ROS\_N} + N}\right)$$

$(AT2)$ cells are depleted due to infection by virus, apoptotic damage by (ROS) secreted by neutrophils (N) and damage due to proinflammatory cytokines at a rate k_{cyt_damage}. $(AT2)$ cells are additionally formed at a rate μ_{AT2} that is dependent on the extent of $(AT2)$ depletion and cleared with a death rate of β_{AT2}.

3 Infected Alveolar Type 2 Cells (I)

$$\frac{dI}{dT} = k_{int}(AT2) \cdot V \left(1 - k_{int(IFN_\beta)} \frac{(IFN_\beta)}{km_{int\_IFN_\beta} + (IFN_\beta)}\right) - \beta_I \cdot I - k_{kill}\left(1 + \frac{(IFN_\beta)}{km_{kill} + (IFN_\beta)}\right) \cdot I \cdot (CTL) - k_{ROS\_damage} \cdot I$$

Infected cells, (I) are formed by infection of $(AT2)$ cells and non-specifically cleared at a rate β_I. Additionally, they are apoptotically cleared by cytotoxic T cells (CTL) and (ROS), and phagocytosed by (DC) and $(M1)$.

4 Healthy Alveolar Type 1 Cells $(AT1)$

$$\frac{dAT1}{dt} = k_{diff\_AT2\_AT1}(AT2) - \beta_{AT1}(AT1) - k_{ROS\_damage}(AT1) - k_{cyt\_damage}(AT1)$$

$$k_{diff\_AT2\_AT1} = k\_basal_{diff\_AT2\_AT1}(1 + k_{\mu}\frac{\delta AT}{km_{\mu} + \delta AT})$$

$$\delta AT = AT1_{basal} + AT2_{basal} - (AT1 + AT2)$$

$(AT1)$ are resistant to infection but can undergo inflammatory cell death mediated by (ROS) and proinflammatory cytokines (k_{cyt_damage}). Additionally, they are formed from differentiation $(AT2)$ cells and undergo non-specific clearance at at a rate β_{AT1}.

5 Damaged Alveolar Type 1 Cells $(DAT1)$

$$\frac{dDAT1}{dt} = k_{ROS\_damage}(AT1) + k_{cyt\_damage}(AT1) - \beta_{dAT1}(DAT1)$$

$DAT1$ are formed by ROS-mediated and cytokine-mediated damage (k_{cyt_damage}) of $AT1$ cells.

6 Damaged Alveolar Type 2 Cells $(DAT2)$

$$\frac{d(DAT2)}{dt} = k_{ROS\_damage}(I + AT2) + k_{cyt\_damage}(AT2) - \beta_{dAT2}(DAT2)$$

$(DAT2)$ are formed by cytokine-mediated damage (k_{cyt_damage}) of $(AT2)$ cells and ROS-mediated damage of $(AT2)$ and (I).

7 Dendritic Cell Matuaration (DC)

$$\frac{dDC}{dt} = \alpha_{DC}\left[k_V log(V) + k_I log(I) + k_D log(DAT1 + DAT2)\right]\left[k_{DC(TNF)}\cdot\left(\frac{(TNF\alpha)}{K_{DC(TNF)} + (TNF\alpha)}\right) + k_{DC(IFN\gamma)}\cdot\left(\frac{(IFN\gamma)}{K_{DC(IFN\gamma)} + (IFN\gamma)}\right) + k_{DC(GM-CSF)}\left(\frac{(GM-CSF)}{K_{DC(GM-CSF)} + (GM-CSF)}\right)\right]\left[\frac{K_{DC(IL10)}}{K_{DC(IL10)} + (IL10)}\right] - \beta_{DC}.(DC) - tr_{DC}$$

Mature (DC) are formed upon recognition of viral particles (V), infected cells (I), and damaged $(AT1)$ and $(AT2)$ cells. (DC) maturation is further induced by $(TNF\alpha)$, $(IFN\gamma)$, $(GM-CSF)$, and inhibited by $(IL10)$. Mature (DC) undergo nonspecific clearance at at a rate β_{DC} and inter-compartmental transport tr_{DC}.

8 Macrophage Activation $(M1)$

$$\frac{dM1}{dt} = \alpha_{M1}\Big[k_V log(V) + k_I log(I) + k_D log(DAT1 + DAT2)\Big]\Big[k_{M1(TNF)}.\left(\frac{(TNF\alpha)}{K_{M1(TNF)} + (TNF\alpha)}\right) +$$

$$k_{M1(IFN\gamma)}.\left(\frac{(IFN\gamma)}{K_{DC(IFN\gamma)} + (IFN\gamma)}\right) + k_{M1(GM-CSF)}.)\left(\frac{(GM-CSF)}{K_{M1(GM-CSF)} + (GM-CSF)}\right)\Big]$$

$$\left[\frac{K_{M1(IL10)}}{K_{M1(IL10)} + (IL10)}\right] - \beta_{M1}.(M1) - tr_{M1}$$

Activated $(M1)$ are formed upon recognition of viral particles (V), infected cells (I), and damaged $(AT1)$ and $(AT2)$ cells. $(M1)$ activation is induced by $(TNF\alpha)$, $(IFN\gamma)$, $(GM-CSF)$, and inhibited by $(IL10)$. Activated $(M1)$ undergo nonspecific clearance at at a rate $\beta_{M1}$and inter-compartmental transport tr_{M1}.

9 Neutrophil Activation (N)

$$\frac{dN}{dt} = \alpha_{N}\Big[k_V log(V) + k_I log(I) + k_D log(DAT1 + DAT2)\Big]\Big[k_{M1(TNF)}.\left(\frac{(TNF\alpha)}{K_{M1(TNF)} + (TNF\alpha)}\right) +$$

$$k_{M1(IFN\gamma)}.\left(\frac{(IFN\gamma)}{K_{DC(IFN\gamma)} + (IFN\gamma)}\right) + k_{M1(GM-CSF)}.)\left(\frac{(GM-CSF)}{K_{M1(GM-CSF)} + (GM-CSF)}\right)\Big]$$

$$ktr_{N[IL-17]} \cdot (\frac{(IL17)}{km_{N\_IL17} + (IL17)}) - \beta_N \cdot N - tr_N$$

Activated (N) are formed upon recognition of viral particles (V), infected cells (I), and damaged $(AT1)$ and $(AT2)$ cells. (N) activation is induced by $(TNF\alpha)$, $(IFN\gamma)$, $(GM-CSF)$, and inhibited by $(IL10)$. Activated (N) undergo nonspecific clearance at at a rate β_N. Activated (N) migration is also induced by $(IL17)$ and undergo inter-compartmental transport tr_N.

10 T-helper 1 (Th1) Cell Activation $(Th1)$

$$\frac{dTh1}{dt} = \alpha_{Th1}\,[DC]\Big[k_{Th1(IL12)}\left(\frac{(IL12)}{K_{Th1(IL12)} + (IL12)}\right)$$

$$\left(1 + k_{Th1(IL12/IL2)}\left(\frac{(IL2)}{K_{Th1(IL12/IL2)} + (IL2)}\right)\right)\left(\frac{K_{Th1(IL10)}}{K_{Th1(IL10)} + (IL10)}\right)\left(\frac{K_{Th1(TGF\beta)}}{K_{Th1(TGF\beta)} + (TGF\beta)}\right) +$$

$$k_{Th1(IFN\gamma)}\left(\frac{(IFN\gamma)}{K_{Th1(IFN\gamma)} + (IFN\gamma)}\right)\left(\frac{K_{Th1(IL10)}}{K_{Th1(IL10)} + (IL10)}\right)\left(\frac{K_{Th1(TGF\beta)}}{K_{Th1(TGF\beta)} + (TGF\beta)}\right)\left(\frac{K_{Th1(IL6)}}{K_{Th1(IL6)} + (IL6)}\right)\Big] +$$

$$k_{Th1(Th17)}.(Th17).\left(\frac{(IL12)}{K_{Th1(Th17)} + (IL12)}\right)\left(\frac{K_{Th1(TGF\beta)}}{K_{Th1(TGF\beta)} + (TGF\beta)}\right) +$$

$$k_{Th1(Treg)}.(Treg).\left(\frac{(IL12)}{K_{Th1(Treg)} + (IL12)}\right) - \beta_{Th1}.(Th1) + tr_{Th1}$$

The production of $(Th1)$ is activated by viral epitope-responsive mature (DC), and further induced by $(IL12)$, $(IL2)$, $(IFN\gamma)$, $(IFN\beta)$ and inhibited by $(IL10)$ and $(TGF\beta)$. Additionally, the ability of $(IFN\gamma)$ and $(IL6)$ to negatively regulate each other's activity is also incorporated. The clearance rate of $(Th1)$ is determined by a nonspecific death/deactivation rate β_{Th1}, and inter-compartmental transport tr_{Th1}.

11 T-helper 17 (Th17) Cell Activation $(Th17)$

$$\frac{dTh17}{dt} = \alpha_{Th17}(DC)\Bigg[k_{Th17(TGF\beta)}.\left(\frac{(TGF\beta)}{K_{Th17(TGF\beta)} + (TGF\beta)}\right) + k_{Th17(IL6)}\left(\frac{(IL6)}{K_{Th17(IL6)} + (IL6)}\right) +$$
$$k\left(\frac{(IL1\beta)}{K_{Th17(IL1\beta)} + (IL1\beta)}\right)\Bigg]\left(\frac{K_{Th17(IL10)}}{K_{Th17(IL10)} + (IL10)}\right)\left(\frac{K_{Th17(IFN\gamma)}}{K_{Th17(IFN\gamma)} + (IFN\gamma)}\right)\left(\frac{K_{Th17(IL2)}}{K_{Th17(IL2)} + (IL2)}\right) -$$
$$k_{Th1(Th17)}.(Th17).\left(\frac{(IL12)}{K_{Th1(Th17)} + (IL12)}\right)\left(\frac{K_{Th1(TGF\beta)}}{K_{Th1(TGF\beta)} + (TGF\beta)}\right) - \beta_{Th17}.(Th17) - tr_{Th17}$$

The production of $(Th17)$ is activated by viral epitope-responsive mature (DC), and further induced by $(TGF\beta)$, $(IL6)$, $(IL1\beta)$ and inhibited by $(IL10)$ and $(IFN\gamma)$. Additionally, the ability of $(IFN\gamma)$ and $(IL6)$ to negatively regulate each other's activity is also incorporated. $(Th17)$ cells can also undergo $(IL12)$-mediated differentiation to $(Th1)$ cells. The clearance rate of $(Th17)$ is determined by a nonspecific death/deactivation rate β_{Th17}, and inter-compartmental transport tr_{Th17}.

12 Cytotoxic T Cell Activation (CTL)

$$\frac{dCTL}{dt} = \alpha_{CTL}\,[DC]\left[1 + \frac{k_{MHCI(IFN\beta)}(IFN\beta)}{km_{MHCI(IFN\beta)} + (IFN\beta)}\right]\Bigg[1 + k_{CTL(IL12)}\left(\frac{(IL12)}{K_{CTL(IL12)} + (IL12)}\right)$$
$$\left(1 + k_{CTL(IL12/IL2)}\left(\frac{(IL2)}{K_{CTL(IL12/IL2)} + (IL2)}\right)\right) +$$
$$k_{CTL(IFN\gamma)}\left(\frac{(IFN\gamma)}{K_{CTL(IFN\gamma)} + (IFN\gamma)}\right)\left(\frac{K_{CTL(IL6)}}{K_{CTL(IL6)} + (IL6)}\right)\Bigg]\left(\frac{K_{CTL(IL10)}}{K_{CTL(IL10)} + (IL10)}\right)\left(\frac{K_{CTL(TGF\beta)}}{K_{CTL(TGF\beta)} + (TGF\beta)}\right)$$
$$- \beta_{CTL}(CTL) - tr_{CTL}$$

The production of (CTL) is activated by viral epitope-responsive mature (DC), and further induced by $(IL12)$, $(IL2)$, $(IFN\gamma)$, $(IFN\beta)$ and inhibited by $(IL10)$ and $(TGF\beta)$. Additionally, the ability of $(IFN\gamma)$ and $(IL6)$ to negatively regulate each other's activity is also incorporated. The clearance rate of (CTL) is determined by a nonspecific death/deactivation rate β_{CTL}, and inter-compartmental transport tr_{CTL}.

13 T regulatory (Treg) Cell Activation $(Treg)$

$$\frac{dTreg}{dt} = \alpha_{Treg}(DC)\Bigg[k_{Treg(IL2)}\left(\frac{(IL2)}{K_{Treg(IL2)}+(IL2)}\right)\left(\frac{K_{Treg(IL17)}}{K_{Treg(IL17)}+(IL17)}\right)\left(\frac{K_{Treg(IL6)}}{K_{Treg(IL6)}+(IL6)}\right)+$$
$$k_{Treg(TGF\beta)}\left(\frac{(TGF\beta)}{K_{Treg(TGF\beta)}+(TGF\beta)}\right)\left(\frac{K_{Treg(IL17)}}{K_{Treg(IL17)}+(IL17)}\right)\left(\frac{K_{Treg(IL6)}}{K_{Treg(IL6)}+(IL6)}\right)\Bigg]$$
$$-k_{Th1(Treg)}.(Treg).\left(\frac{(IL12)}{K_{Th1(Treg)}+(IL12)}\right)-\beta_{Treg}(Treg)-tr_{Treg}$$

The production of $(Treg)$ is activated by viral epitope-responsive mature (DC), and further induced by $(TGF\beta)$ and $(IL2)$ and inhibited by $(IL17)$ and $(IL6)$. $(Treg)$ cells can also undergo $(IL12)$-mediated differentiation to $(Th1)$ cells. The clearance rate of $(Treg)$ is determined by a nonspecific death/deactivation rate β_{Treg}, and inter-compartmental transport tr_{Treg}.

14 Tumor necrosis factor α $(TNF\alpha)$

$$\frac{dTNF\alpha}{dt} = \alpha_{TNF}.\Bigg[\alpha_{TNF(basal)}+\alpha_{TNF(dAT1)}(DAT1)+\alpha_{TNF(I)}\cdot I+\alpha_{TNF(dAT2)}(DAT2)$$
$$\alpha_{TNF(M1)}(M1)+\alpha_{TNF(Th1)}(Th1)+\alpha_{TNF(Th17)}(Th17)\Bigg]-\beta_{TNF}(TNF\alpha)-tr_{TNF\alpha}$$

$(TNF\alpha)$ is secreted by $(DAT1)$, $(DAT2)$, (I), $(M1)$, $(Th1)$ and $(Th17)$. $(TNF\alpha)$ additionally has a basal non-specific production rate $\alpha_{TNF}.\alpha_{TNF(basal)}$, a clearance rate β_{TNF} and inter-compartmental transport $tr_{TNF\alpha}$.

15 Interluekin-6 (IL-6) $(IL6)$

$$\frac{dIL6}{dt} = \alpha_{IL6}\Bigg[\alpha_{IL6(basal)}+\alpha_{IL6(dAT1)}.(DAT1)+\alpha_{IL6(I)}\cdot I+\alpha_{IL6(dAT2)}(DAT2)+\alpha_{IL6(M1)}(M1)+$$
$$\alpha_{IL6(Th17)}(Th17)+\alpha_{IL6(Neu)}(N)+\alpha_{IL6(I)}\cdot I\Bigg]-\beta_{IL6}(IL6)-tr_{IL6}$$

$(IL6)$ is secreted by $(DAT1)$, $(DAT2)$, (I), $(M1)$, (N) and $(Th17)$. $(IL6)$ additionally has a basal non-specific production rate $\alpha_{IL6}.\alpha_{IL6(basal)}$, a clearance rate β_{IL6} and inter-compartmental transport tr_{IL6}.

16 Interleukin-1β (IL-1β) $(IL1\beta)$

$$\frac{dIL1\beta}{dt} = \alpha_{IL1\beta}\Bigg[\alpha_{IL1\beta(basal)}+\alpha_{IL1\beta(dAT1)}.(DAT1)+\alpha_{IL1\beta(I)}\cdot I+\alpha_{IL1\beta(dAT2)}(DAT2)$$
$$+\alpha_{IL1\beta(M1)}(M1)+\alpha_{IL1B(DC)}(DC)+\alpha_{IL1\beta((I)}\cdot I\Bigg]-\beta_{IL1\beta}(IL1\beta)-tr_{IL1\beta}$$

$(IL1\beta)$ is secreted by $(DAT1)$, $(DAT2)$, (I), $(M1)$ and (DC). $(IL1\beta)$ additionally has a basal non-specific production rate $\alpha_{IL1\beta}.\alpha_{IL1\beta(basal)}$, a clearance rate $\beta_{IL1\beta}$ and inter-compartmental transport $tr_{IL1\beta}$.

17 Interferon γ (IFN$\boldsymbol{\gamma}$) $(IFN\gamma)$

$$\frac{dIFN\gamma}{dt} = \alpha_{IFN\gamma}\left[\alpha_{IFN\gamma(basal)} + \alpha_{IFN\gamma(DC)}.(DC) + \alpha_{IFN\gamma(Th1)}(Th1) + \alpha_{IFN\gamma(CTL)}(CTL)\right] - \beta_{IFN\gamma}(IFN\gamma) - tr_{IFN\gamma}$$

$(IFN\gamma)$ is secreted by $(Th1)$, (CTL) and (DC). $(IFN\gamma)$ additionally has a basal non-specific production rate $\alpha_{IFN\gamma}.\alpha_{IFN\gamma(basal}$ a clearance rate $\beta_{IFN\gamma}$ and inter-compartmental transport $tr_{IFN\gamma}$.

18 Type I Interferons $(IFN\beta)$

$$\frac{dIFN\beta}{dt} = \alpha_{IFN\beta}\left[\alpha_{IFN\beta(basal)} + \alpha_{IFN\beta(I)} \cdot I + \alpha_{IFN\beta(DC)}(DC)\right] - \beta_{IFN\beta}(IFN\beta) - tr_{IFN\beta}$$

$(IFN\beta)$ is secreted by (I) and (DC). $(IFN\beta)$ additionally has a basal non-specific production rate $\alpha_{IFN\beta}.\alpha_{IFN\beta(basal)}$, a clearance rate $\beta_{IFN\beta}$ and inter-compartmental transport $tr_{IFN\beta}$.

19 Interleukin-2 (IL-2) $(IL2)$

$$\frac{dIL2}{dt} = \alpha_{IL2}\left[\alpha_{IL2(basal)} + \alpha_{IL2(DC)}(DC) + \alpha_{IL2(Th1)}(Th1)\right] - \beta_{IL2}(IL2) - tr_{IL2}$$

$(IL2)$ is secreted by (DC) and $(Th1)$. $(IL2)$ additionally has a basal non-specific production rate $\alpha_{IL2}.\alpha_{IL2(basal)}$, a clearance rate β_{IL2} and inter-compartmental transport tr_{IL2}.

20 Interluekin-12 (IL-12) $(IL12)$

$$\frac{dIL12}{dt} = \alpha_{IL12}\left[\alpha_{IL12(basal)} + \alpha_{IL12(DC)}(DC) + \alpha_{IL12(M1)}(M1)\right] - \beta_{IL12}(IL12) - tr_{IL12}$$

$(IL12)$ is secreted by (DC) and $(M1)$. $(IL12)$ additionally has a basal non-specific production rate $\alpha_{IL12}.\alpha_{IL12(basal)}$, a clearance rate β_{IL12} and inter-compartmental transport tr_{IL12}.

21 Interleukin-17 (IL-17) $(IL17)$

$$\frac{dIL17}{dt} = \alpha_{IL17}\left[\alpha_{IL17(basal)} + \alpha_{IL17(Th17)}(Th17) + \alpha_{IL17(CTL)}(CTL)\right] - \beta_{IL17}(IL17) - tr_{IL17}$$

$(IL17)$ is secreted by (CTL) and $(Th17)$. $(IL17)$ additionally has a basal non-specific production rate $\alpha_{IL17}.\alpha_{IL17(basal)}$, a clearance rate β_{IL17} and inter-compartmental transport tr_{IL17}.

22 Interluekin-10 (IL-10) $(IL10)$

$$\frac{dIL10}{dt} = \alpha_{IL10}\left[\alpha_{IL10(basal)} + \alpha_{IL10(Treg)}(Treg)\right] - \beta_{IL10}(IL10) - tr_{IL10}$$

$(IL10)$ is secreted by $(Treg)$. $(IL10)$ additionally has a basal non-specific production rate $\alpha_{IL10}.\alpha_{IL10(basal)}$, a clearance rate β_{IL10} and inter-compartmental transport tr_{IL10}.

23 Transforming growth factor β (TGF-β) $(TGF\beta)$

$$\frac{dTGF\beta}{dt} = \alpha_{TGF\beta}\left[\alpha_{TGF\beta(basal)} + \alpha_{TGF\beta(Treg)}(Treg) + \alpha_{TGF\beta(Th17)}(Th17)\right] - \beta_{TGF\beta}(TGF\beta) - tr_{TGF\beta}$$

$(TGF\beta)$ is secreted by $(Treg)$ and $(Th17)$. $(TGF\beta)$ additionally has a basal non-specific production rate $\alpha_{TGF\beta}.\alpha_{TGF\beta(basal)}$, a clearance rate $\beta_{TGF\beta}$ and inter-compartmental transport $tr_{TGF\beta}$.

24 Granulocyte macrophage-colony stimulating factor (GM-CSF) $(GM{-}CSF)$

$$\frac{d(GM-CSF)}{dt} = \alpha_{GM-CSF}\left[\alpha_{GM-CSF(basal)} + \alpha_{GM-CSF(Th1)}(Th1) + \alpha_{GM-CSF(M1)}(M1) + \alpha_{GM-CSF(Th17)}(Th17)\right] - \beta_{GM-CSF}(GM-CSF) - tr_{GM-CSF}$$

$(GM-CSF)$ is secreted by $(M1)$, $(Th17)$ and $(Th1)$. $(GM-CSF)$ additionally has a basal non-specific production rate $\alpha_{GM-CSF}.\alpha_{GM-CSF(basal)}$, a clearance rate β_{GM-CSF} and inter-compartmental transport tr_{GM-CSF}.

25 C-reactive Protein

$$\frac{dCRP_{extracellular}}{dt} = k_{CRP\_secretion}V_{m\_Prot\_synth} \cdot vol_{liver}(IL6_c) + k_{tr(CRP)}vol_{liver} - k_{deg\_CRP}(CRP_{extracellular})$$

$$\frac{dCRP_{blood}}{dt} = k_{basal\_CRP} - k_{tr(CRP)} - k_{deg\_CRP}(CRP_{blood})$$

CRP is produced in the liver $((CRP_{exrtracellular}))$ and is induced by liver concentrations of $(IL6)$. $(CRP_{exrtracellular})$ is tranported to blood at an inter-compartmental transit rate $k_{tr(CRP)}$. (CRP_{blood}) is also basally produced at a rate k_{basal_CRP}. Both $(CRP_{exrtracellular})$ and (CRP_{blood}) are cleared at a rate k_{deg_CRP} from their respective compartments.

26 Surfact Protein-D (SPD)

$$\frac{dSPD}{dt} = k_{basal\_SPD} + \alpha_{SPD(AT2)}(DAT2) + \alpha_{SPD(AT1)}(DAT1) + \alpha_{SPD(CTL\_I)}k_{kill}\left(1 + \frac{IFN_\beta}{km_{kill} + IFN_\beta}\right) \cdot I \cdot (CTL) - k_{tr(SPD)}(SPD)$$

(SPD) is released by $(DAT1)$, $(DAT2)$ and the cytotoxic clearance of (I) by (CTL) in the alveolar compartment. (SPD) has an inter-compartmental transit rate of $k_{tr(SPD)}$.

27 Ferritin (FER)

$$\frac{dFER}{dt} = k_{basal\_FER} + \alpha_{FER(AT1)}(DAT1) + \alpha_{FER(AT12)}(DAT2) + \alpha_{FER(CTL\_I)}k_{kill}\left(1 + \frac{IFN_\beta}{km_{kill} + IFN_\beta}\right) \cdot I \cdot (CTL) - k_{tr-FER}$$

(FER) is released by $(DAT1)$, $(DAT2)$ and the cytotoxic clearance of (I) by (CTL) in the alveolar compartment. (FER) has an inter-compartmental transit rate of k_{tr-FER}.

28 Immune Cell Transport

$$\frac{dIC_c}{dt} = tr_{IC}(IC) - \beta_{IC_c}(IC_c)$$

$$tr_{IC} = k_{tr\_IC}\Big(IC - IC_c\Big)$$

29 Cytokine Tranport

$$\frac{dCytokine_c}{dt} = tr_{cytokine} - \beta_{cytokine_c}(Cytokine_c)$$

$$tr_{cytokine} = k_{tr\_cytokine}(Cytokine)$$

30 Biomarker Tranport

$$\frac{dSPD\_c}{dt} = k_{tr\_SPD}(SPD) - \beta_{SPDc}(SPD\_c)$$

$$\frac{dFER\_c}{dt} = k_{tr\_FER}(FER) - \beta_{FERc}(FER\_c)$$
